# Diaryl Sulfide Derivatives as Potential Iron Corrosion Inhibitors: A Computational Study

**DOI:** 10.3390/molecules26206312

**Published:** 2021-10-19

**Authors:** Morad M. El-Hendawy, Asmaa M. Kamel, Mahmoud M. A. Mohamed, Rabah Boukherroub, Jacek Ryl, Mohammed A. Amin

**Affiliations:** 1 Department of Chemistry, Faculty of Science, New Valley University, Kharga 72511, Egypt; asmaa.kamel@scinv.au.edu.eg (A.M.K.); mmhm802004@yahoo.com (M.M.A.M.); 2 University of Lille, CNRS, Centrale Lille, Université Polytechnique Hauts-de-France, UMR 8520-IEMN, F-59000 Lille, France; rabah.boukherroub@univ-lille.fr; 3 Institute of Nanotechnology and Materials Engineering, Gdansk University of Technology, Narutowicza 11/12, 80-233 Gdansk, Poland; 4 Department of Chemistry, College of Science, Taif University, P.O. Box 11099, Taif 21944, Saudi Arabia

**Keywords:** diaryl sulfides, DFT, MC simulation, corrosion inhibitor, dapsone

## Abstract

The present work aimed to assess six diaryl sulfide derivatives as potential corrosion inhibitors. These derivatives were compared with dapsone (4,4′-diaminodiphenyl sulfone), a common leprosy antibiotic that has been shown to resist the corrosion of mild steel in acidic media with a corrosion efficiency exceeding 90%. Since all the studied compounds possess a common molecular backbone (diphenyl sulfide), dapsone was taken as the reference compound to evaluate the efficiency of the remainder. In this respect, two structural factors were examined, namely, (i) the effect of replacement of the S-atom of diaryl sulfide by SO or SO_2_ group, (ii) the effect of the introduction of an electron-withdrawing or an electron-donating group in the aryl moiety. Two computational chemical approaches were used to achieve the objectives: the density functional theory (DFT) and the Monto Carlo (MC) simulation. First, B3LYP/6-311+G(d,p) model chemistry was employed to calculate quantum chemical descriptors of the studied molecules and their geometric and electronic structures. Additionally, the mode of adsorption of the tested molecules was investigated using MC simulation. In general, the adsorption process was favorable for molecules with a lower dipole moment. Based on the adsorption energy results, five diaryl sulfide derivatives are expected to act as better corrosion inhibitors than dapsone.

## 1. Introduction

Corrosion is a serious problem, as it threatens our life. Corrosives components can cause damage to not only metals but also to the human digestive and respiratory tracts, eyes, and skin. Using coating materials as inhibitors can lengthen the life and usability of metallic components, machinery, products, etc. Continuous advances in computer efficiency and computational chemistry software allowed examining different types of corrosion inhibitors. Density Functional Theory (DFT) and Monte Carlo (MC) simulation methods have recently been accepted as fast and powerful tools for estimating the relative corrosion inhibition activities of various molecules [1,2]. Since corrosion processes and their prevention by organic inhibitors is a very active research area, many researchers have focused their studies on this subject. Many researchers reported that some physicochemical and electronic properties of the organic inhibitor’s functional groups, steric effects, electronic density of donor atoms, and orbital character of donating electrons affect the inhibition efficiency of metals [3,4].

Several organic and inorganic corrosion inhibitors are expensive, toxic, and harmful to the environment, restricting their use. Currently, researchers shifted their focus on expired drugs as a safe alternative for protecting metals if they meet the following criteria [5]: (i) the chemical composition of the drug must have active centers such as N, O, and S atoms, (ii) it must be less hazardous and environmentally friendly, and (iii) it can be easily produced and purified. Several research studies reported different types of drugs (melatonin, cephapirin, tramadol, etc.) as corrosion inhibitors for various metals [6,7,8,9,10]. The findings indicated that these drugs form an insoluble complex on the metal surface, protecting it from corrosion.

Recently, Danaee et al. [6] studied experimentally and theoretically the inhibitory effect of an oxethazaine, 2,2′-(2-hydroxyethylimino)bis[N-(alphaalphadimethylphenethyl)-N-methylacetamide] drug on corrosion of mild steel in 1 M HCl. Theoretical results were found to be compatible with the experimental findings. Additionally, Sachin et al. demonstrated that L-Dopa, a drug used to treat Parkinson’s disease, significantly minimized the corrosion of mild steel in both HCl and H_2_SO_4_. The findings indicate that inhibition results from the formation of a non-porous organic film on the metal surface, and the adsorption follows the Langmuir isotherm [7].

Dohare et al. [8] demonstrated the inhibitory effect of tramadol for protecting mild steel in acidic media by electrochemical impedance spectroscopy, potentiodynamic polarization, surface analysis (SEM and AFM), and DFT methods. The influence of cephapirin drugs on the corrosion of carbon steel was examined by weight loss and electrochemical methods [10]. EIS studies revealed that the inhibition process occurred through charge transfer. The quantum chemical calculations proved that cephapirin is a suitable corrosion inhibitor against carbon steel. Overall, research studies revealed that drugs as corrosion inhibitors are the best choice for protecting metals and lead to the management of expired drug waste.

Diaryl sulfide compounds and their sulfoxide and sulfone derivatives have long attracted the interest of researchers as effective corrosion inhibitors [11,12,13,14,15,16]. In 1980, Abdelaal et al. demonstrated the anticorrosive inhibition of diphenyl sulfide (Ph_2_S), diphenyl sulfoxide (Ph_2_SO), and diphenyl sulfone (Ph_2_SO_2_) against tin and cadmium in acidic solutions [11]. The adsorption of inhibitors obeyed a Tomkin isotherm. The inhibiting effect decreased in the series: Ph_2_S > Ph_2_SO_2_ > Ph_2_SO. Earlier, the inhibiting efficiencies of some sulfoxides with aliphatic chains or aromatic rings were investigated in an acidic medium [12]. The anticorrosive activity followed the sequence: dibenzyl sulfoxide > di-*n*-butyl sulfoxide > di-p-tolyl sulfoxide > diphenyl sulfoxide > tetramethylene sulfoxide > dimethyl sulfoxide. The anticorrosive activity was is influenced by the electron density of the sulfur atom and their rate of reduction to sulfide. Recently, James and Lalgudi reported in their patent that a metal-appended dichloro-diphenyl sulfone anti-corrosion additive could reduce the corrosion susceptibility of carbon steel and other metal substrates when added to topcoats or primer [13]. The influence of the addition of organic sulfur compounds (mercaptan, diphenyl sulfide, and diphenyl disulfide) was studied on the corrosion caused by elemental sulfur in synthetic naphtha [14]. The study indicated that diphenyl disulfide was the best corrosion inhibitor of elemental sulfur.

Although the mode of action of drugs within the mammalian body differs from their anticorrosive mechanism on a metal surface, a common factor uniting the two functions is the distinct electronic and geometric structures that facilitate chemical and physical attachment to a biological receptor or metal surface. For example, dapsone (4,4′-diamino diphenyl sulfone), the common antibiotic drug for treating leprosy, has proved its anticorrosive merit for mild steel in acidic media (1 M HCl and 0.5 H_2_SO_4_) [15]. The superiority of the anticorrosive activity of dapsone bearing the diphenyl sulfide backbone [15] motivated us to explore more compounds with the same molecular backbone. The finding revealed that inhibition occurs through drug adsorption on the metal surface without modifying the corrosion mechanism. Recently, Singh et al. reacted the expired drug, dapsone, with benzaldehyde and salicylaldehyde to produce dapsone-benzaldehyde and dapsone-salicylaldehyde, respectively [16]. The resulting products exhibited maximum corrosion inhibition efficiencies of 95.67% and 94.23% for mild steel at a concentration of 0.219 mM in an acidic medium. The addition of a tiny amount of KI further increased their efficiencies up to 99.03% and 97.98%, respectively. Additionally, diphenyl sulfides and their sulfoxide and sulfone analogs proved their merit as excellent corrosion inhibitors [11,12,13,14]. The common structural factor among all these molecules is diphenyl sulfide, which reflects its superiority in anti-corrosion activity. 

**Figure 1:**
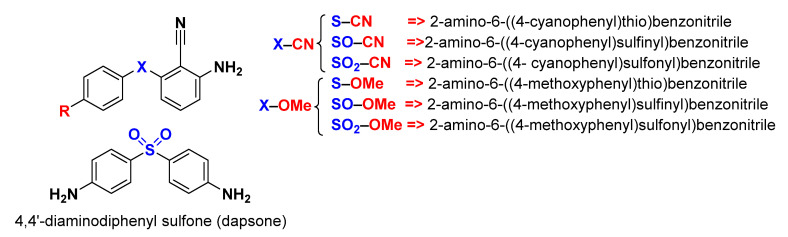
Chemical structures of investigated compounds with their IUPAC names as well as nicknames. The common molecular structure of the seven compounds is diphenyl sulfide.

Sulfur-containing compounds, such as sulfides, sulfoxides, and sulfones, constitute a large portion of pharmaceutical drugs that possess various biological activities [17]. For example, Chan et al. assessed the antiviral activity against HIV-1 of a series of 2-amino-6-arylthiobenzonitriles and their sulfoxides and sulfones derivatives [18]. Based on the above, we chose six molecules from the long list of this series of 2-amino-6-arylthiobenzonitriles and their sulfoxide and sulfone derivatives, as shown in Figure 1, to examine their anticorrosive behavior on steel. We divided the studied molecules into two families to facilitate the discussion of results: X–CN and X–OMe, where X is S, SO, or SO_2_ group. In this respect, the DFT method and MC simulation were applied in the absence and presence of iron metal, respectively. Several quantum chemical descriptors of the isolated inhibitors (Table 1) were calculated to study their global reactivity and selectivity toward other species. Furthermore, the adsorption descriptors and the mode of adsorption on the iron surface were calculated by MC simulation.

## 2. Computational Details

All DFT calculations were performed using Gaussian 16 suite [19]. In this context, the B3LYP/6-311+G(d,p) method was utilized for geometry optimization in the aqueous phase, followed by frequency calculations to confirm that the optimized geometry was not a saddle point. The polarizable continuum model (PCM) using the integral equation formalism variant (IEFPCM) was employed for modeling the aqueous phase [20]. Molecular properties related to the reactivity and selectivity of the compounds were estimated following Koopmans’s theorem [21] that depends on the energy of the frontier molecular orbitals. According to DFT-Koopmans’s theorem [21,22], the ionization potential (I) and electron affinity (A) can be approximated as the negative values of the E_HOMO_ and E_LUMO_, respectively. The quantum chemical descriptors and the mathematical forms used for their estimation are collected in Table 1.

**Table 1:** The mathematical forms for estimating quantum chemical descriptors.

Descriptor	Mathematical Form
Ionization potential (*I*)	*I = −E_HOMO_*
Electron affinity (*A*)	*A = −E_LUMO_*
Energy gap (Δ*E*)	Δ*E* *= E_LUMO_* *−E_HOMO_*
Electronegativity (*χ*) [23,24]	
Chemical hardness (*η*) [23,24]	
Global electrophilicity index (*ω*) [25]	
The number of electrons transferred (ΔN) from the inhibitor to the iron surface. The work function of metal is the work function of iron [26]	
The energy associated with a backing donation	

Monte Carlo simulations [27] were conducted using the Adsorption Locator module [28] as implemented in BIOVIA Materials Studio 2017 [29] to locate the low energy adsorption sites of the potential corrosion inhibitors on the Fe surface. All components, such as clean iron surface, inhibitor, and the acidic medium, were optimized first using the Condensed-phase Optimized Molecular Potentials for Atomistic Simulation Studies (COMPASS) force field [30]. Ewald and atom-based summation approaches were employed to calculate the electrostatic and van der Waals energies, respectively. The Fe(110) crystal is the most stable facet; thus, it was selected for the simulation [31]. An amount of 1 M HCl was commonly used in the experiment as an acidic medium; thus, 5 HCl/278 H_2_O were used to represent its effect on adsorption mode [32]. To provide an appropriate surface for the interaction with the inhibitors, a 10 × 10 supercell was built with a 30 Å thick vacuum slab.

## 3. Results and Discussion

### 3.1. Quantum Chemical Study

The geometry, electron density distribution of HOMO and LUMO, and ESP are presented in Figure 1. The findings indicate a bent molecular geometry of all studied molecules. The substitution of the S atom of diaryl sulfides by SO or SO_2_ groups impacts the geometry as seen from the C–S bond length, <C–S–C bond angle, and <C–S–C–C dihedral angle (Table 2). For example, ongoing from S–CN to SO–CN and SO_2_–CN, the bond length increases by 0.05 and 0.02 Å, and the bond angle changes by −7° and + 1°, respectively. A tremendous and steady decrease in dihedral angles was observed ongoing from S–CN to SO–CN and SO_2_–CN. Even small changes in the structural parameters can produce significant changes in the predicted energy and other molecular properties [33].

**Figure 2:**
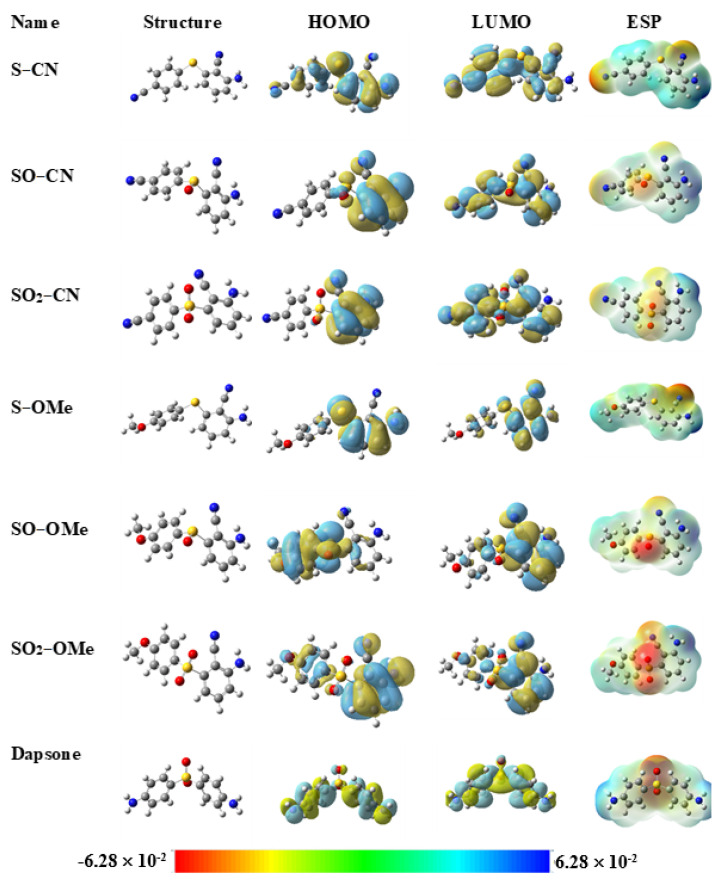
The geometry, frontier molecular orbital electron density distribution, and electrostatic potential map of the investigated molecules. The colored bar of MEP indicates the positive and negative potentials.

The frontier molecular orbital topologies of the studied inhibitors are illustrated in Figure 2. The HOMOs for all studied inhibitors are π-type molecular orbitals. The HOMO surface of the X−CN corrosion inhibitors is essentially localized over the disubstituted aryl moiety, indicating that it is electron-rich and may be involved in electron donation to the metal surface. Further, the LUMO plot shows a delocalization of the lowest vacant molecular orbitals over the entire skeleton, suggesting that the low-lying vacant orbitals of the inhibitors could be available for back-donation. A different situation is observed in the case of X−OMe inhibitors. For example, the HOMO surface of SO−OMe is mainly localized on the sulfoxide group and the mono-substituted aryl moiety, while the LUMO is shifted toward the other moiety. This behavior is a feature of the intramolecular charge transfer where the HOMO and LUMO are localized on the donor and acceptor moieties, respectively. Because of the symmetrical geometry of dapsone, the HOMO and LUMO electron density are delocalized over the entire molecular skeleton.

The molecular electrostatic potential (MEP) of a molecule represents its electrophilic and nucleophilic reactivities, according to the colored bar in Figure 2. For example, the color between red and green is susceptible to electrophilic attack; in contrast, green and blue are accessible to nucleophilic attack. At first glance, the most negative potential (red color) is located around the oxygen atoms of the SO and SO_2_ groups, especially in the case of SO–OMe and SO_2_–OMe, reflecting the role of the methoxy group at the para position to provide these atoms with excess electron density.

The global reactivity parameters of the studied inhibitors are gathered in Table 3. According to the FMO theory, the E_HOMO_ and E_LUMO_ are associated with the electron-donating and electron-accepting capacities of the molecule, respectively [34]. Therefore, a molecule with a higher E_HOMO_ and a lower E_LUMO_ is expected to adsorb strongly to the metallic surface. The results indicate that E_HOMO_ and E_LUMO_ steadily are reduced ongoing from the diaryl sulfides to diaryl sulfones. Thus, the electron-donating capacity decreases when the electron-accepting capabilities of the inhibitor increase in this direction. Similarly, the second structural factor of replacing X−CN with X−OMe gives the same trend. Therefore, the net inhibition score cannot be derived from both parameters.

Since the hardness of a molecule is a function of the energy gap ΔE, we discuss the changes in the ΔE values. ΔE is a function of reactivity of the inhibitor molecule towards the adsorption on a metallic surface. With a decrease in ΔE, the reactivity of the molecule increases, which leads to improved inhibition efficiency of the molecule. According to Table 3, the value of ΔE steadily decreases when the S atom is replaced by SO and SO_2_ either for X−CN or X−OMe molecules. Moreover, in general, X-CN molecules are more reactive and less rigid than X-OMe. On the other hand, the reactivity of dapsone is lower at least by 0.5 eV than those of the rest of the molecules due to the symmetrical structure.

The dipole moment of molecules can reflect their ability to protect the metal surface [35,36,37]. The dipole moment of X−CN molecules is higher than that of X−OMe molecules. It was reported that a molecule with a lower dipole moment favors its accumulation on the surface layer, leading to higher inhibition efficiency [38].

Molecular volume (MV) illustrates the possibility of the inhibitor covering the metal surface. A molecule that has a considerable MV value has the highest protection to the metal surface. There is no specific order when replacing the S atom with the SO or SO_2_ group or when replacing the cyano group with the methoxy one, but the volume of the molecule depends on the degree of twisting in the molecule. S−CN is the highest in MV and SO_2_−OMe is the lowest one.

ΔN is usually used to indicate the amount and direction of electron flow within molecular systems. Positive values of ΔN for all studied molecules imply the electron donation from the inhibitor to the metal surface atoms. The value of ΔN is comparable (~2 electrons) for all the tested compounds except for dapsone, which is 1.73 e. Furthermore, the back-donation can be evaluated by the value of ∆E_b-d_. The negative sign of ∆E_b-d_ indicates that back-donation to the inhibitor is energetically favorable. All molecules have comparable values ~−0.55 eV. The donation and back-donation processes strengthen the adhesion of the inhibitor molecules to the iron surface.

### 3.2. Monte Carlo (MC) Simulations

**Figure 3:**
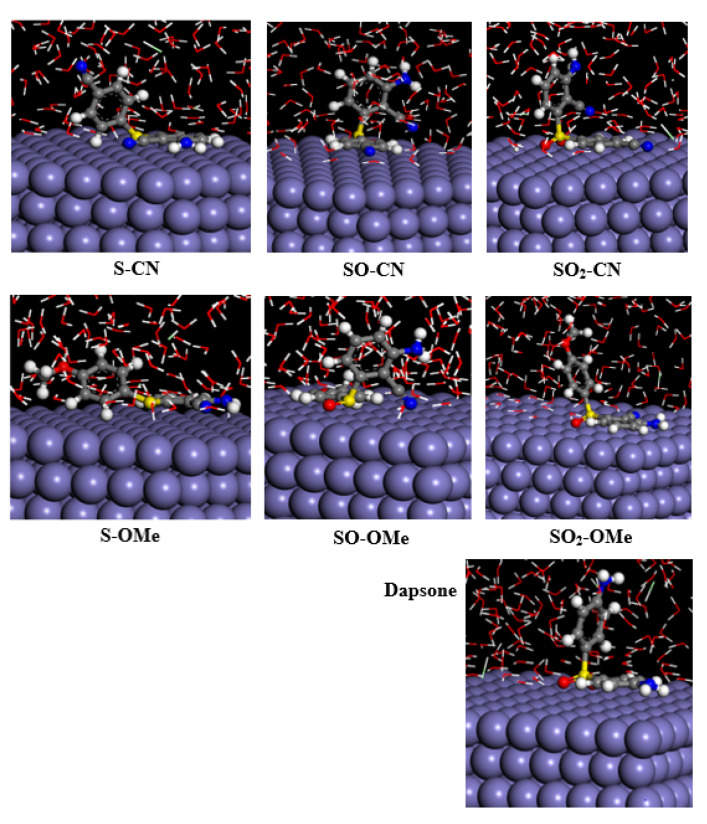
Side views of the most stable configurations of the adsorbed inhibitors on Fe(110) surface in acidic medium (1 M HCl).

The studied molecules contain two different aryl moieties: mono-substituted and disubstituted aryl moieties. Since the studied inhibitors have bent molecular geometry, both moieties cannot be adsorbed together on the surface. Figure 3 illustrates the most stable configurations for their adsorption on the Fe(110) surface. The findings indicate that the disubstituted aryl moiety of diaryl sulfides (S–CN and S–OMe) is horizontally loaded onto the iron surface, while the mono-substituted aryl one is almost perpendicular to it. This means that replacing the electron-withdrawing group (CN) with an electron-donating one (OMe) does not affect the adsorption mode. The preference for sticking the disubstituted moiety to the surface is attributed to its inclusion of more donor atoms than the mono-substituted one.

The mode of adsorption was changed after replacing the S atom of the diaryl sulfide with the SO group, as the mono-substituted aryl moiety with the SO moiety was loaded onto the surface instead of disubstituted aryl one. Additionally, it was observed that the oxygen atom of the SO fragment carries the highest negative charge (~−0.94). Thus, one can expect how such molecules adsorb on the metallic surface; first, the SO fragment attacks the surface, then the most coplanar moiety attaches to the surface. A similar situation can be expected in SO_2_ molecules, because the oxygen atoms carry the highest negative atomic charges. For example, introducing an OMe group instead of the CN one in the SO–CN molecule does not affect adsorption, as the mono-substituted moiety and SO fragment adsorbed together on the iron surface. This is attributed to the smaller twisting angle between the mono-substituted aryl moiety and the SO fragment by 10° compared to the disubstituted counterpart for both molecules.

An unusual mode of adsorption is observed in the case of SO_2_-based molecules, as it depends on the nature of the R-group, whether it is an electron withdrawer or an electron donor. For example, in the case of the SO_2_–CN molecule, the dihedral angles between the mono and disubstituted aryl moieties with the SO_2_ fragment are 180 and 145 degrees, respectively. Thus, the more planar mono-substituted aryl ring has a higher tendency to adsorb on the surface, as indicated from the MC simulation (Figure 3). On the other hand, the two moieties of the SO_2_–OMe molecule tilt by approximately 5 degrees with the SO_2_ fragment, i.e., the exact extent of coplanarity. Therefore, the second factor (atomic charge) becomes the playmaker of the adsorption process. Accordingly, it is found that the disubstituted aryl ring adheres to the surface, because it possesses more than one donor atom ready to interact with the surface.

Based on the above, one can conclude that two factors influence the adsorption mode: the geometry of the inhibitor and its atomic charges. This finding could help the rational design of new corrosion inhibitors. In the case of dapsone, the adsorption mode is similar to the other sulfone molecules.

The MC adsorption parameters are listed in Table 4. The meaning of these parameters is discussed in our previous paper [37]. At first glance, all the adsorption energies of the studied systems are negative, indicating the automatic adsorption process. Additionally, the high negative adsorption energy suggests strong bonding between the adsorbates and the substrate [37]. The replacement of the S atom of the diaryl sulfide by SO or SO_2_ group impacts the adsorption energy. For example, replacing the sulfur atom of the S–CN molecule with an SO group increased the adsorption energy by 24 kcal/mol, while replacing it with an SO_2_ group decreased it by 19 kcal/mol. Additionally, replacing the S atom of the S–OMe molecule with SO or SO_2_ group decreased/increased the adsorption energy by 7 and 6 kcal/mol, respectively. These changes are attributed to the selection of the inhibitor to the mode of adsorption on the Fe surface, as discussed above. Interestingly, the adsorption energy of dapsone is higher than all other studied compounds except for SO–CN. Therefore, one can expect that the studied sulfides, sulfoxides (except SO–CN), and sulfones would have inhibition efficiency exceed 91% of dapsone [15]. 

On the other hand, the effect of insertion of the methoxy group instead of the cyano group is clearer, where the energy required for adsorption decreases in this direction. In such a case, the methoxy group increases the electron density on the phenyl ring and the sulfur atom, facilitating the interaction with the surface. Therefore, the inhibitors can be arranged according to the strength of their interaction with the surface as follows: SO–OMe > S–OMe > SO_2_–OMe = SO_2_–CN > S–CN > dapsone > SO–CN.

By comparing the average adsorption energies of the inhibitors (−3835 kcal/mol) with their average desorption energies (dE_ad_/dN_i_) (−143 kcal/mol), it was found that the difference between them is significant, which indicates that the adsorption process is irreversible and heavily favored.

## 4. Conclusions

The present work aimed to study the possibility of utilizing six non-nucleoside reverse transcriptase inhibitors of HIV-1 (diaryl sulfide derivatives) as anticorrosive materials for steel. Since dapsone has the same molecular backbone (diphenyl sulfide) as all studied molecules, and its inhibition efficiency has been tested experimentally, it was used here as a reference compound to evaluate the efficiency of the investigated compounds. In this regard, the effect of two structural factors on the inhibition ability was studied: (i) the effect of replacing the sulfide group of diaryl sulfide with sulfoxide and sulfonyl ones, and (ii) the effect of R-substituent by replacing the cyano group with methoxy. Two computational approaches were employed to achieve this goal: DFT and MC simulation. Based on the findings, one can conclude the following points:

- The DFT geometry of the studied molecules is not flat and has a bent molecular shape, which leads to incomplete molecular adsorption on the surface.
- Two factors control the adsorption mode on the iron surface: the extent of coplanarity of aryl moiety with the X-group and the value of individual negative atomic charges.
- The dipole moment of the studied molecules correlates well with their adsorption ability. A molecule with a lower dipole moment has better adsorption on the iron surface.
- Although the X–CN molecules are more reactive and less rigid than X–OMe molecules, the latter have a stronger ability for adsorption because of the high electron-donating ability of the methoxy group.
- Based on the adsorption study, all the studied compounds, except for SO–CN, show higher inhibition efficiency than dapsone. Accordingly, we nominate these inhibitors as effective iron surface corrosion inhibitors.

## Figures and Tables

**Table 2:** Bond length, bond angle, and dihedral angle between sulfur atoms and sulfur-adjacent carbon atoms in the studied inhibitors.

	S–CN	SO–CN	SO_2_–CN	S–OMe	SO–OMe	SO_2_–OMe	Dapsone
C_M_–S; C_D_–S (Å) *	1.79; 1.79	1.84; 1.84	1.81; 1.82	1.79; 1.79	1.82; 1.84	1.79; 1.82	1.79; 1.79
<C_M_–S–C_D_ (°)	104	97	105	104	98	105	106
<C_M_–C_M_–S–C_D_ (°)	139	97	82	89	88	84	90
<C_D_–C_D_–S–C_M_ (°)	142	83	72	2	82	73	90

* The symbols M and D in C_M_ and C_D_ refer to the carbon atom in the mono-substituted and disubstituted aryl moiety.

**Table 3:** The DFT global reactivity descriptors of the studied inhibitors.

Molecule	E_HOMO_ (eV)	E_LUMO_ (eV)	∆E (eV)	DM. (D)	MV (cm^3^/mol)	η (eV)	χ (eV)	ω (eV)	∆E_b-d_ (eV)	∆N (e)
S−CN	−6.28	−1.85	4.43	12.27	214	2.22	−4.07	3.73	−0.56	2.06
SO−CN	−6.39	−2.00	4.39	14.41	206	2.19	−4.19	4.01	−0.55	2.11
SO_2_−CN	−6.48	−2.29	4.19	12.89	195	2.10	−4.39	4.59	−0.53	2.25
S−OMe	−6.19	−1.75	4.44	6.09	186	2.22	−3.97	3.54	−0.56	2.04
SO−OMe	−6.35	−1.92	4.43	9.84	210	2.21	−4.13	3.86	−0.55	2.08
SO_2_−OMe	−6.43	−2.14	4.29	10.47	161	2.15	−4.28	4.28	−0.54	2.17
Dapsone	−5.93	−1.00	4.93	6.38	175	2.47	−3.47	2.44	−0.62	1.73

**Table 4:** The output adsorption descriptors (kcal/mol) for inhibitor/Fe(110) systems in acidic medium.

	Total Energy	Adsorption Energy	Rigid Adsorption Energy	Deformation Energy	*d*E_ad_/*d*N_i_		
					Inh	H_2_O	HCl
S–CN	−3791	−3826	−4023	197	−148	−11	−9
SO–CN	−3760	−3802	−3993	191	−135	−12	−8
SO_2_–CN	−3799	−3845	−4040	195	−162	−14	−7
S–OMe	−3823	−3851	−4046	195	−146	−12	−9
SO–OMe	−3825	−3858	−4054	196	−125	−13	−9
SO_2_–OMe	−3808	−3845	−4040	196	−151	−12	−7
Dapsone	−3795	−3816	−4007	191	−137	−11	−7

## Data Availability

All of the research data are available from the authors.
